# Highlight: After the Bottleneck—How a Tiny Group of Macaques Founded a Thriving Population on Mauritius

**DOI:** 10.1093/gbe/evv051

**Published:** 2015-03-28

**Authors:** Danielle Venton

Once upon a time the island of Mauritius held no mammals. But as this small island, 2,000 km off the coast of Africa in the Indian Ocean, became an important stop on the trade route between the East and Europe, it was visited frequently by both Dutch and Portuguese sailors. And it was likely one of these sea-faring salty dogs who brought Mauritius its first nonhuman mammal: the “crab-eating” or cynomolgus macaque, likely from Java, originally as pets.

“The founding population was probably between 4 and 12 at the most,” says Antoine Blancher, an immunologist at the Toulouse University Hospital in France (CHU de Toulouse). “But they grew very fast and became important invaders. The poor farmers who were trying to cultivate sugar cane were totally desperate because the macaques destroyed all their efforts. The farmers eventually abandoned the island and the macaque population just exploded.”

Thus the seeds were planted for an interesting experiment: how would the descendants fare? Would harmful mutations accumulate in the population? Or, might selection win out over genetic drift, keeping the population relatively healthy? A recent whole-genome analysis of a selection of these monkeys, completed by Osada et al. (2015) reveal that these macaques retained a surprising amount of genetic diversity, and have enjoyed fairly good health, during their 400 or so years on Mauritius.

As a more practical matter, for medical researchers the cynomolgus macaque is a key animal used to test vaccines, immunosuppressive drugs for organ transplants and other treatments. “For experimental immunologists these animals are very precious,” Blancher says. Genetically they are quite close to humans yet they are still allowed to be used in experiments (in the United States the government recently outlawed the use of chimpanzees in research, following a recommendation by the National Institutes of Health). This particular kind of macaque is especially attractive to researchers, as they are free of the herpes B virus—a potentially fatal disease for humans.

To survey the polymorphic diversity within the Mauritian cynomolgus macaques (*Macaca fasicularis*) the team extracted DNA from blood samples of six wild-caught macaques collected for a different research project. They then sequenced the genomes at the Beijing Genomics Institute in Shenzhen, China.

The results were a surprise for the research team. “Despite this severe population bottleneck, the Mauritian macaques were quite diverse—about as polymorphic as the global human population,” Blancher says.

And as for the accumulation of deleterious mutations? None were seen—an observation consistent with recent theoretical and experimental work in humans finding that recent demographic changes do not strongly affect the genetic load of a population. Here, it seems the push of selection has balanced the pull of genetic drift.

“One force is eliminating these deleterious mutations and the other one is promoting their accumulation and the balance is relatively neutral,” says Blancher. “They do not accumulate dangerous mutation as was assumed before, in the case of most Mauritius animals they are perfectly healthy. This is very good news, I think, for species conservationists.”

If the starting population is genetically quite heterogeneous, he says, a healthy population can be maintained starting from just a few individuals.

The observed level of polymorphism also makes these monkeys well suited to some areas of research. “Ignoring the degree of polymorphism in your study animals is very dangerous,” Blancher says. “If you validate your work with only a few animals you can be totally puzzled by the response when the treatment is used in humans.”

“This is important work,” says Zhenxin Fan, a genetics researcher at Sichuan University in China, who has previously sequenced Tibetan macaques. “The smaller genetic diversity of this population than Malaysian cynomolgus macaques is an advantage in biomedical studies and we need to know their genetic diversity and demographic history.”

In the future, however, he'd like to see an investigation of the very recent demographic history of this population, which the relatively small sample size used in this study could not infer.

Osada et al. (2015) are now working on finding an association between cynomolgus macaques polymorphism and the capacity of an individual to fight viral infection, specifically simian immunodeficiency virus, the monkey equivalent of human immunodeficiency virus.

“For us the next step is finding the polymorphisms that increase the capacity to resist infection,” he says. “But I think people can now look at the polymorphism involved with other physical characteristics of these animals. Many studies can start now.”
